# The Role of Sleep Quality and Sleepiness in the Relationship Between Cognitive Flexibility and Fatigue

**DOI:** 10.1007/s11126-025-10135-9

**Published:** 2025-04-05

**Authors:** Ozge Ozkutlu, Ozgu Inal Ozun

**Affiliations:** Gulhane Faculty of Physiotherapy and Rehabilitation, University of Health Sciences, Ankara, Turkey

**Keywords:** Sleep, Sleepiness, Fatigue, Cognitive Flexibility

## Abstract

This study aimed to determine the pathways linking cognitive flexibility to fatigue through the serial mediation effect of sleep quality and sleepiness in 564 healthy adults using various scales, including the Pittsburgh Sleep Quality Index, Epworth Sleepiness Scale, Chalder Fatigue Scale, and Cognitive Flexibility Inventory. Analysis was performed using two mediating variables (sleep quality and sleepiness) with age and gender as covariates. The simple mediation effect was evaluated using the Causal Step. The indirect effect was examined using bootstrap method and Sobel test. The total and direct effects of cognitive flexibility on fatigue were both found to be statistically significant. The research model revealed three significant indirect effects: cognitive flexibility affecting fatigue through sleep quality, cognitive flexibility affecting fatigue through sleepiness and cognitive flexibility affecting fatigue through both sleep quality and sleepiness. Additionally, serial mediation analysis showed that the relationship between cognitive flexibility and fatigue is sequentially mediated by sleep quality and sleepiness (B = -0.064, SE = 0.013, 95% CI: -0.070 to -0.017). All effects were confirmed through the 95% confidence intervals, which did not include zero, and with significant p values. These findings highlight the role of sleep quality and sleepiness as serial mediators of the relationship between cognitive flexibility and fatigue. The study's major contribution is to provide insight on the independent and serial mediating role of sleep quality and sleepiness in the relationship between cognitive flexibility to fatigue.

**Supplementary Information:**

The online version contains supplementary material available at 10.1007/s11126-025-10135-9.

## Introduction

Sleep is a vital physiological requirement that individuals spend one-third of their lives on, enabling them to perform their activities and roles appropriately [1]. Sleep deprivation has been shown to have a major impact on cognitive performance, with some studies claiming that it affects global cognitive skills such as alertness and attention, while others focus on higher cognitive skills such as perception, memory, and executive functions [2]. It reveals that sleepiness can increase reaction times, alter emotional states, and influence decision-making. The effects depend on the cognitive ensemble required for the task [3]. Sleep deprivation significantly affects executive function, specifically cognitive flexibility (CF), which involves the adaptation of cognitive control resources to changes in events. This is particularly relevant for tasks requiring executive control to achieve goals [4, 5].

In some social, occupational, and clinical settings, the phrases "sleepy" and "tired" are used interchangeably. Fatigue can occur with or independently of sleepiness. Although excessive daytime sleepiness is sometimes accompanied by physical and mental exhaustion, sleepiness is defined as a physically felt desire to sleep. Sleepiness can cause performance to slow down or cease completely. Although the performance can persist for an extended period, it may become difficult to complete and exhaustion may become apparent during the performance. While most patients who report excessive daytime sleepiness also express fatigue, not everyone with fatigue has excessive daytime sleepiness. There is a domino effect when sleepiness invades daily life, as the consequences of sleepiness can lead to both fatigue and cognitive dysfunction [6].

Fatigue was considered a multidimensional condition with various origins, such as hormonal changes, stress, unhealthy lifestyle, and disrupted sleep patterns. Previous studies examining the effects of fatigue on cognition specifically report difficulties with executive functions [7, 8]. Only a limited number of studies have investigated the effects of fatigue on CF. These studies suggest a relationship between fatigue and decreased CF [9–12]. In the study of Plukaard and his colleagues (2015) it was found that induced fatigue in students reduced the ability to switch between tasks of equal difficulty, slowed response, and increased switch costs, impacted CF [13].

Quantity of sleep has been found to be a significant mediator of cognitive function [14]. Poor sleep efficiency and both short and lengthy sleep durations are linked to an increased risk of cognitive impairment as well as worse performance on executive function tasks such as working memory, attention, and CF [15]. Sleeping for the recommended amount of hours is essential for maintaining cognitive abilities including CF, which in turn affects an employee's capacity to complete tasks and overall performance [16]. Optimal cognitive functioning is fundamental to performance within many work environments (exp. active military personnel, aviation pilots, air traffic controllers) [17]. A person's ability to do (1) basic, low-salience tasks or (2) high-salience tasks requiring flexible attentional and working memory allocation with crucial or safety-sensitive outcomes should be especially concerning in the context of sleep restriction [17]. Furthermore, even though they don't do safety–critical tasks, elite athletes also frequently depend on their cognitive ability to perform at their best under time- and environment-constrained conditions (e.g., concentration, executive functioning, decision making) [18]. Therefore, it is necessary to understand the relationship between concepts related to cognitive function and sleep.

Although fatigue, CF, and sleep interact through a complex network, the precise mechanisms underlying these relationships remain unclear. Previous studies have examined various aspects of this network, such as the effects of sleep deprivation on CF and the role of sleep disorders in fatigue [5, 19]. However, no study has explicitly explored the mediating role of sleep quality and sleepiness in the relationship between CF and fatigue. While existing research focuses on individual associations, it overlooks the potential mediating pathways through which sleep-related factors could influence CF and fatigue. This gap in the literature limits our understanding of how these elements interact. To address this deficiency, the present study aimed to investigate whether sleep quality and sleepiness act as serial mediators in the relationship between CF and fatigue. We hypothesize that sleep quality and sleepiness will act as serial mediators between CF and fatigue.

## Methods

### Participants

The study involved healthy adults aged 18–65, recruited through social media, and assessed for compliance with inclusion and exclusion criteria. Participants had full access to the evaluation form's content if they met the criteria. To reach the required sample size, the cluster sampling method was preferred. For observational studies with large population size that involve logistic regression in the analysis, taking a minimum sample size of 500 is necessary to derive the statistics that represent the parameters [20]. Informed consent was obtained from all participants prior to the beginning of the study. Participants were provided with detailed information regarding the purpose, duration, potential risks, and benefits of the study, and it was emphasized that participation was entirely voluntary. All participant data was kept confidential and used solely for research purposes. This study was performed in line with the principles of the Declaration of Helsinki. Approval was granted by the Ethics Committee of Gulhane Scientific Research (2023–240). The inclusion criteria for the study are being 18–65 years old, having normal or corrected vision, being literate, being able to use a smartphone, and not having a diagnosed neurological or psychiatric disease. Participants who were diagnosed with a sleeping disorder, were pregnant, used psychotropic medication (antidepressants), and had a history of cancer were excluded from the study.

### Evaluation

Researchers utilized a sociodemographic information form to record patient demographics and physical findings, including age, weight, height, and gender.

#### Sleep Quality

The sleep quality of the patients was evaluated with the "Pittsburgh Sleep Quality Index ", which has been shown to be valid and reliable [21]. This index is a subjective self-report scale that evaluates one-month sleep quality and sleep disturbance. It has seven subcategories on subjective sleep quality, sleep latency, sleep duration, sleep efficiency, sleep disorders, sleep medication use, and daytime dysfunction. The response of each subscale is scored as 0–3. The total score range varies between 0–21.

#### Sleepiness

The sleepiness of the patients was evaluated with the "Epworth Sleepiness Scale", which has been shown to be valid and reliable [22]. The questionnaire had a high level of internal consistency as measured by Cronbach's alpha (> or = 0.86) This scale involves eight items on a person's sleepiness. It was scored on a scale of 0, no chance of dozing, to 3, high chance of dozing. The overall score ranges from 0 to 24, with an increase in the score indicating increasing sleepiness.

#### Fatigue

Fatigue was evaluated with the "Chalder Fatigue Scale", which has been shown to be valid and reliable [23]. The scale’s Cronbach's alpha = 0.863 showed great internal consistency, while intraclass correlation coefficient = 0.76 indicated good test–retest reliability. The scale has two subsections: physical and mental fatigue. There are 11 items in total in the scale, 7 items assessing physical fatigue and 4 items assessing mental fatigue. Each item is answered with a 4-point Likert type: "less than usual", "as much as usual", "more than usual", "much more than usual". The physical fatigue subsection score varies between 0–21 points, and the mental fatigue subsection score varies between 0–12 points. The overall fatigue score is obtained by adding up the scores obtained from the physical fatigue and mental fatigue sections.

#### Cognitive Flexibility

Participants' CF was evaluated with the "Cognitive Flexibility Inventory", which has been shown to be valid and reliable [24]. This inventory was designed to measure people's ability to produce alternative, harmonious, appropriate, and balanced thoughts in difficult situations. It consists of twenty items and has two subscales. As the score obtained from the scale increases, CF also increases.

## Statistical Analysis

Continuous data were analyzed with means and standard deviations, and categorical data with frequencies and percentages. Kolmogorov–Smirnov was used to determine the normality of continuous data. The relationships between the variables were evaluated with Pearson, Spearman (correlation includes BMI) and Point-biserail correlation analyses (correlation includes gender), and the following cut-off points were used for the interpretation of the correlation coefficients; 0.00–0.10 negligible, 0.10–0.39 weak, 0.40–0.69 moderate, 0.70–0.89 strong and 0.90–1.00 very strong relationship [25]. The simple mediation effect using a single mediator variable was evaluated with the Causal Step approach suggested by Baron and Kenny [26] and analyzed using Model4 with the SPSS Process ver.4.2 macro program developed by Hayes [27]. The significance of the indirect effect was examined using the bootstrap technique and Sobel test [28]. Serial Multiple Mediation Analysis using two mediating variables was analyzed using Model6 with the SPSS Process ver.4.2 macro program. Age and gender were included in the model as covariates. The significance of the indirect effect was evaluated with the lower and upper limits of the 95% confidence intervals and effect coefficients obtained with 5000 bootstrap samples using the bootstrap technique [29]. Data was analyzed using the IBM SPSS statistical package, version 23.0. The significance level was set as 0.05. The post-hoc analysis conducted using G*Power for (v 3.1.5; Franz Faul, Kiel University, Kiel, Germany) for linear multiple regression confirmed that with a sample size of 564, an effect size of 0.064, and an alpha level of 0.05, the statistical power of the regression model was 99%.

## Results

A total of 564 people, 190 men (33.7%) and 374 women (66.3%), participated in the study. The average age of the participants was 32.45 ± 12.18 year (range: 19–64). The average Body Mass Index (BMI) of the participants was determined as 24.17 ± 4.35.

## Correlation Analysis

Correlations for all study variables were presented in Online Resource.1. Fatigue was negatively correlated with CF (r = −0.264, p < 0.01) and positively correlated with sleepiness and poor sleep quality (r = 0.249, p < 0.01; r = 0.446, p < 0.01 respectively). CF was negative correlated with sleepiness and poor sleep quality (r = −0.203, p < 0.01; r = −0.110; p = 0.009). Sleepiness is positively correlated with poor sleep quality (r = 0.192, p < 0.01). Older persons were less likely to feeling fatigued (r = −131, p = 0.002), have poor sleep quality (r = −0.097; p = 0.021), while they had greater CF (r = 0.108, p = 0.011). Sleepiness, sleep quality, age and gender, which had a statistically significant relationship between fatigue and CF, were included in simple and serial multiple mediation analyzes. CF was used as independent variable and fatigue was used as dependent variable.

## Simple Mediation Model

First, sleep quality and sleepiness were uniquely tested with the 4 hypotheses of the causal step approach to determine whether they mediate the relationship between CF and fatigue.

**Fig. 1:**
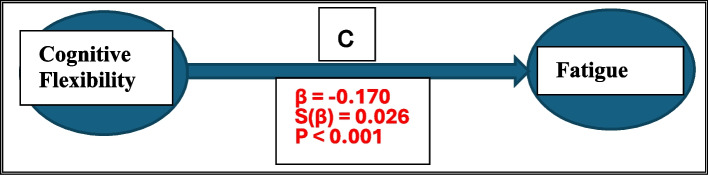
Direct Effect Model

According to the first hypothesis of the causal approach, there must be a statistically significant relationship between the dependent variable and the independent variable. It was found that CF significantly impacts fatigue (p < 0.001) as indicated by the regression coefficient (β = −0.170) and standard error (s(β) = 0.026) obtained from modeling the relationship between CF as the independent variable and the fatigue as the dependent variable. Figure 1 illustrates the direct effect model.

**Fig. 2:**
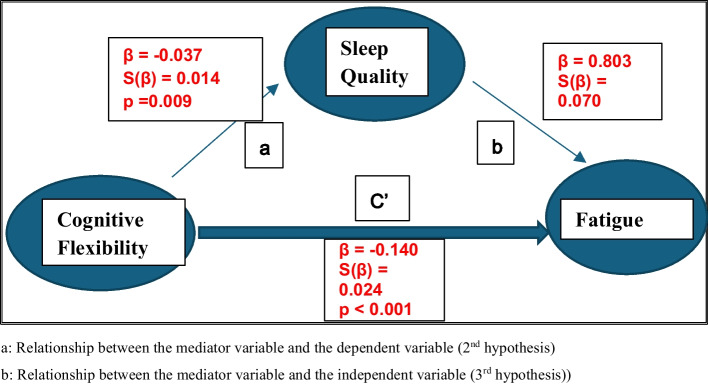
Simple Mediation Model in which Sleep Quality as the Mediator

**Fig. 3:**
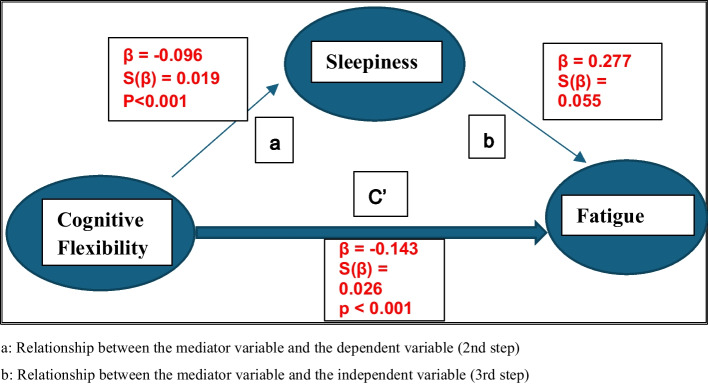
Simple Mediation Model in which Sleepiness as the Mediator

The second and third hypothesis of the Causal Step Approach are shown In Fig. 2 for sleep quality and in Fig. 3 for sleepiness. Accordingly, it was seen that both CF and fatigue have a significant effect on the sleep quality as the mediator variable (β = −0.037, p = 0.009; β = 0.803, p < 0,001 respectively). CF and fatigue also significantly effected sleepiness as the mediator variable (β = −0.096, p < 0,001; β = 0.277, p < 0,001). (Fig. 3).

According to the 4th hypothesis, the coefficient of the independent variable (Fig. 1—section c) in the model should be greater than the coefficient of the independent variable in the model with the independent variable and the mediator variable (c' in Figs. 2 and 3). It was observed that the absolute value of the regression coefficient between CF and fatigue (0.170) obtained in the model in Fig. 1 was greater than the absolute value of the regression coefficient (0.140) obtained in the model in Fig. 2 (0.170–0.140 = 0.030). Similarly, the absolute value of the regression coefficient between CF and fatigue (0.169) in Fig. 1 was bigger than the absolute value of the regression coefficient (0.143) in Fig. 3 (0.169–0.0143 = 0.026). Accordingly, sleep quality and sleepiness may have mediator role in the relationship between CF and fatigue.

Table 1 shows the direct, indirect, and total effects of CF on fatigue when sleep quality was the mediator. The total and direct effect of CF on fatigue (−0.170 ± 0.026 and −0.140 ± 0.024) was found to be statistically significant (p < 0.001). The confidence intervals of the indirect effect were accepted as significant because the lower (−0.055) and upper (−0.007) limits of the 95% confidence intervals do not include zero according to the bootstrap method (Table 1). The indirect effect of CF on fatigue through sleep quality was also found to be statistically significant (z = −5.718, SE = 0.025, p < 0.001) in Sobel test. It has been revealed that CF can negatively affect the fatigue variable through sleep quality and sleep quality may have partial mediating role.

**Table 1:** Direct, indirect and total effects

Fatigue	Direct effect β ± S(β)	Indirect effect β ± S(β)	%95 Confidence Interval ^a, b^ Lower–Upper Bound	Total effect β ± S(β)	%95 Confidence Interval ^a,b^ Lower–Upper Bound
**Cognitive flexibility** **(Independent variable**)	−0.140 ± 0.024 (p < 0.001)	−0.030 ± 0.012	(−0.055)-(−0.007)	−0.170 ± 0.026 (p < 0.001)	(−0.118) - (−0.264)

a: Obtained by sample of 5.000 Bootstrap

b: 95% Bias corrected Confidence Interval

^*^Sleep Quality as mediating role

Table 2 shows the direct, indirect, and total effects of CF on fatigue when sleepiness was the mediator. The total and direct effect of CF on fatigue (−0.169 ± 0.026 and −0.143 ± 0.026) was found to be statistically significant (p < 0.001). The confidence intervals of the indirect effect were accepted as significant because the lower (−0.043) and upper (−0.012) bounds of the 95% confidence intervals do not include zero according to the bootstrap method (Table 2). The indirect effect of CF on fatigue through sleepiness was also found to be statistically significant (z = −3,567, SH = 0,007, p < 0,001) in Sobel test. It has been revealed that CF can negatively affect fatigue through sleepiness and sleepiness may have partial mediating role.

**Table 2:** Direct, indirect and total effects

Fatigue	Direct Effect β ± S(β)	Indirect Effect β ± S(β)	%95 Confidence Interval ^a,b^ Lower Upper Bound	Total Effect β ± S(β)	%95 Confidence Interval ^a,b^ Lower Upper Bound
Cognitive Flexibility (Independent Variable)	−0.143 ± 0.026 (p < 0.001)	−0.026 ± 0.008	(−0.043)- (−0.012)	−0.169 ± 0.026 (p < 0.001)	(−0.221)- (−0.118)

a: Obtained by sample of 5,000 Bootstrap

b: 95% Bias corrected Confidence Interval

^*^Sleepiness as mediating role

## Serial Multiple Mediation Model

**Fig. 4:**
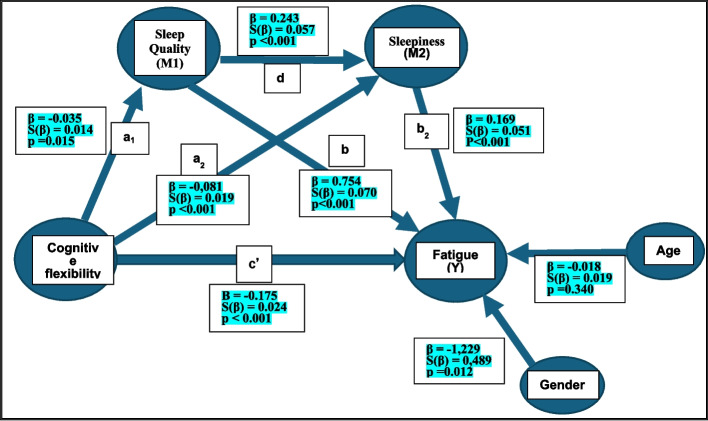
Serial Multiple Mediation Model

Figure 4 shows the serial multiple mediation model in which CF (X) affects fatigue (Y) through the mediating variables of sleep quality (M1) and sleepiness (M2) with age and gender as covariates.

The table containing all data on direct effects is included in Online Resources 2. The direct total effect of CF on fatigue (c) (β: −0.112, S(β) = 0.024, p < 0.001) was found to be negative and statistically significant. According to the Bootstrap technique, the lower (−0.159) and upper (−0.065) bounds of the 95% confidence intervals were evaluated as significant because they did not contain zero.

CF had a negative and statistically significant effect on the sleep quality (mediator variable M1) (β: −0,035, S(β) = 0,014, p = 0.015). CF had a negative and statistically significant effect on the sleepiness (mediator variable M2) (β: −0.081, S(β) = 0.019, p < 0.001). The first mediator variable (Sleep Quality) had a positive and statistically significant effect on the second mediator variable (Sleepiness) (β:0.243, S(β) = 0.057, p < 0.001). The table containing all data on direct effects is included in Online Resources 2.

Direct effect standard value (B = −0,175, SH = 0,024, 95%CI = [−0.159, −0.065], p < 0.001) and total effect standard value (B = −0,239, SH = 0,026, 95%CI = [−0.205, −0.102], p < 0.001) was found to be statistically significant as the p-value was less than 0.001 and the Boostrap 95% confidence intervals did not include zero (Table 3).

**Table 3:** Serial multiple mediation model: indirect and total effects

Effects c = c′ + a_1_b_1_ + a_2_b_2_ + a1d1b2	Standard Effect Value (B)	Boostrap Standard Error	95% confidence interval ^a,b^	
			Lower Bound	Upper Bound
Total Effect (c)	−0.239	0.026 p < 0.001	−0.205	−0.102
Direct Effect (c')	−0.175	0.024 p < 0.001	−0.159	−0.065
1.Indirect Effect X → M1 → Y a_1_b_1_	−0.041	0.011	−0.049	−0.005
2.Indirect Effect X → M2 → Y a_2_b_2_	−0.021	0.006	−0.027	−0.004
3.Indirect Effect X → M1 → M2 → Y a_1_d_1_b_2_	−0.002	0.001	−0.004	- < 0.001
Total Indirect Effects c—c′: a_1_b_1_ + a_2_b_2_ + a_1_d_1_b_2_	−0.064	0.013	−0.070	−0.017

a: Obtained by sample of 5,000 Bootstrap

b: 95% Bias corrected Confidence Interval

The research model reveals three indirect effects and a total indirect effect:

The first indirect effect (a_1_b_1_) of CF on fatigue was through sleep quality, and this effect was found to be negative (CF → sleep quality → fatigue; B = −0.041, SE = 0.011) as the Boostrap 95% confidence intervals did not include zero (Table 3).

The second indirect effect (a_2_b_2_) of CF on fatigue was through sleepiness, and this effect was also found to be negative (CF → sleepiness → fatigue; B = −0.021, SE = 0.006) as the Boostrap 95% confidence intervals did not include zero (Table 3).

The third indirect effect (a_1_*d_1_*b_2_) of CF on fatigue was mediated by sleep quality and sleepiness and this effect was also found to be negative (CF → sleep quality → sleepiness → fatigue; B = −0.002, SE = 0.001). The indirect effect was found to be statistically significant as the Boostrap 95% confidence intervals did not include zero (Table 3).

The sum of the above three indirect effects shows the total indirect effect of CF on fatigue (serial mediation effect (c—c′: a_1_b_1_ + a_2_b_2_ + a_1_d_1_b_2_). This effect was found to be negative (B = −0.064, SE = 0.013). The serial mediation effect was found to be statistically significant as the Boostrap 95% confidence intervals (95% CI = [−0.070, −0.017]) did not include zero (Table 3).

## Discussion

The study explored the mediating role of sleep quality and sleepiness in the relationship between CF and fatigue, revealing both serial multiple and separate mediation effects.

## Cognitive Flexibility and Fatigue

In our study, the role of sleep quality and sleepiness in the relationship between CF and fatigue was investigated. CF requires inhibition (or deactivate) the previous perspective and loading into working memory (or activation) a different perspective. It is in this sense that CF requires and builds on inhibitory control and working memory [30]. One of the most important reasons for the decrease in response inhibition was shown to be mental fatigue. This might be attributed to a slowing of the inhibition process, a delay in the assessment of visual cues, or a reduction in the availability of attentional resources [31]. Mental fatigue affects cognition both in chronic conditions, such as in patients with chronic fatigue syndrome [32], or acute conditions as in the healthy young population [31]. Most studies on the relationship between cognition and fatigue focus on mental fatigue [33, 34]. Studies on both physical and mental fatigue and cognition has been conducted, particularly in conditions such as multiple sclerosis [35] or stroke [36], where both mental and physical fatigue play a significant role. In addition, maximal exercise has been found to have a negative impact on cognitive performance in a study conducted on recreational athletes. [37]. In our study, we employed a questionnaire that assessed both physical and mental fatigue, which is important since it was demonstrated both fatigues can affect CF [38]. The relationship between CF and fatigue has both been demonstrated by two studies conducted on healthy students [13, 39]. Both studies demonstrate that CF declines as mental fatigue increases. The neurophysiological explanation of the link between CF and fatigue originates from the prefrontal cortex. The behavioral results showed that task switching was affected by time on target effects, mainly when working memory processes were needed to control task switching [40]. In conclusion, CF and fatigue appear to intersect, especially in working memory and response inhibition.

## Sleep Quality as a Mediator

Firstly, we demonstrated the mediating role of sleep quality in the relationship between CF and fatigue. Adequate sleep time is not enough to complete sleep quality; at the same time, effective sleep must be ensured from different aspects [21]. There are two distinct stages of sleep: non-rapid eye movement (NREM) and rapid eye movement (REM). Within the sleep cycle, REM sleep is also significant. It is believed that flexible, or "fluid," cognitive processes—which call for a certain neurophysiological environment—are essential to problem solving and creative abilities. Dreaming while in REM sleep is linked to the strength of weak links in cognitive networks, which in turn leads to creative and abstract thought. REM sleep may give a distinct style of problem solving compared with waking and NREM [41]. Many features of sleep appear to affect cognition differently. Therefore, we used an instrument that assesses sleep quality under many subheadings in our study. In the study of Anderson and colleagues, it was revealed that CF and sleep quality were related in a healthy population aged between 50 and 85 [42]. In the other hand, the effects of sleep quality and fatigue have been discussed in many different populations. Its effect on the healthy young population was discussed in the study of Pastier and colleagues. The strongest relationship was found in between mental fatigue and sleep quality. In addition, participants who reported poor sleep quality also reported greater levels of physical and mental fatigue. Similarly, state fatigue (present mood) was linked to worse sleep quality [43]. Considering, we might say that sleep quality is a characteristic that should not be overlooked in the relationship between CF and fatigue, as demonstrated in our study.

## Sleepiness as a Mediator

Secondly, we demonstrated the mediating role of sleepiness in the relationship between CF and fatigue. Sleepiness and fatigue are difficult to distinguish from each other, even as terms [44]. The study of Hossain and colleagues revealed that subjective sleepiness and subjective fatigue are independent manifestations of sleep disorders [45]. The effects of sleepiness and fatigue have been investigated together in many pathologies [46]. The cognitive function that occurs in situations related to fatigue and sleepiness has also been investigated. The study of Neu et al. found that both fatigue and sleepiness-associated conditions can significantly impair cognitive functioning [47]. Another study which conducted on shift workers revealed that nocturnal sleepiness negatively impacts CF, specifically set inhibition, causing decreased accuracy and increased perseverative errors, and hindering the ability to reactivate previously performed tasks [48]. Considering, we might say that sleepiness is a characteristic that should not be overlooked in the relationship between CF and fatigue, as demonstrated in our study.

## Sleep Quality and Sleepiness as Serial Mediators

Thirdly, this study found a significant pathway of CF → sleep quality → sleepiness → fatigue. This model suggests that the serial relationship between sleep quality and sleepiness mediates the relationship between CF and fatigue. There is a strong connection between sleep quality and sleepiness. This association tends to be related to sleep deprivation. Sleep deprivation has been associated with a loss of CF due to "feedback blunting," which means that feedback on behavioral outcomes has less relevance—and so is less effective at motivating behavior adjustment under changing conditions. This aspect of sleep deprivation-related decline in performance appears to be unique from vigilant attention deficiencies, and it provides a particularly major problem for fatigue risk management [5]. It seems essential to consider sleep quality and sleepiness in the relationship between fatigue and CF.

In conclusion, this study provides empirical evidence for the pathways linking CF to fatigue. More specifically, the current study's major contribution is to provide insight on the independent and accumulative mediating role of sleep quality and sleepiness in the relationship between CF to fatigue. It is thus possible to illustrate that this relationship is partially due to the link between sleep quality and sleepiness. This study draws out the implication for the importance of sleep quality and sleepiness when investigating the mechanisms through which CF relates to fatigue. Cognitive interventions which can be affected by fatigue can benefit from strategies to improve sleep quality and reduce sleepiness. Our research discovered that to remain cognitively flexible without fatigue, it is necessary to prioritize sleep quality.

The study has limitations due to its non-clinical, homogeneous community sample, which may limit its generalizability to other clinical populations and specific populations. The gender ratio was 66.3%, and the study was cross-sectional, preventing the establishment of causality. Sleep quality and sleepiness were evaluated subjectively, and the generalizability of the findings may be limited to the specific study population and setting. The study also acknowledges a limitation regarding directionality. CF was analyzed as the independent variable. However, previous research suggests a bidirectional relationship, where fatigue may also affect CF. This ambiguity complicates the interpretation of their interrelationship and highlights the need for caution in drawing definitive conclusions, as other confounding factors may also play a role. Accepting fatigue as a dependent variable aligns with significant research examining its effects on cognitive functioning and performance, thereby contributing to the broader discourse in the field. Future studies should consider reversed models that account for the potential influence of fatigue on CF. By investigating both directions of the relationship, researchers could gain a more comprehensive understanding of how these two variables influence each other. Such studies could also help inform intervention strategies that target both CF and fatigue, offering more holistic approaches to treatment.

## Supplementary Information

Below is the link to the electronic supplementary material.

Supplementary file1 (DOCX 16 KB)

Supplementary file2 (DOCX 16 KB)

## Data Availability

Data supporting the findings of this study will be made available upon reasonable request.
