# Supplementary material for: The Role of Sleep Quality and Sleepiness in the Relationship Between Cognitive Flexibility and Fatigue
Source: Psychiatr Q. 2025 Apr 5;96(4):735–49. doi: 10.1007/s11126-025-10135-9 (PMC12647325; doi:10.1007/s11126-025-10135-9)
Supplement: Supplementary file 1 — Supplementary file1 (DOCX 16 KB) [file 11126_2025_10135_MOESM1_ESM.docx]

**Appendix A. Correlations Between Study Variables**

|  | | **Fatigue** | **Cognitive Flexibility** | **Sleepiness** | **Sleep Quality** | **Body Mass Index** | **Age** | **Gender** |
| --- | --- | --- | --- | --- | --- | --- | --- | --- |
| **Fatigue** | **r** |  | -.264 | .249 | .446 | .057 | -.131 | -.165 |
|  | **p** |  | **.000** | **.000** | **.000** | .173 | **.002** | **.000** |
| **Cognitive Flexibility** | **r** |  |  | -.203 | -.110 | .024 | .108 | .169 |
|  | **p** |  |  | **.000** | **.009** | .569 | **.011** | **.000** |
| **Sleepiness** | **r** |  |  |  | .192 | .101 | -.033 | -.112 |
|  | **p** |  |  |  | **.000** | **.017** | .435 | **.007** |
| **Sleep Quality** | **r** |  |  |  |  | -.020 | -.097 | -.030 |
|  | **p** |  |  |  |  | .638 | **.021** | .477 |
| **Body Mass Index** | **r** |  |  |  |  |  | -.036 | -.030 |
|  | **p** |  |  |  |  |  | .394 | .479 |
| **Age** | **r** |  |  |  |  |  |  | .335 |
|  | **p** |  |  |  |  |  |  | **.000** |
