# Supplementary material for: The Role of Sleep Quality and Sleepiness in the Relationship Between Cognitive Flexibility and Fatigue
Source: Psychiatr Q. 2025 Apr 5;96(4):735–49. doi: 10.1007/s11126-025-10135-9 (PMC12647325; doi:10.1007/s11126-025-10135-9)
Supplement: Supplementary file 2 — Supplementary file2 (DOCX 16 KB) [file 11126_2025_10135_MOESM2_ESM.docx]

Appendix B. Serial Multiple Mediation Model Direct Effect

|  | **M1 (Sleep Quality)** | | | **M2 (Sleepiness)** | | | **Y (Fatigue)** | | | |
| --- | --- | --- | --- | --- | --- | --- | --- | --- | --- | --- |
|  | **β** | **S(β)** | **p** | **β** | **S(β)** | **p** | **β** | **S(β)** | **p** | **%95 CI^a,b^**  **Lower-Upper Bound** |
| **(X) Cognitive Flexibility** | -0.035 | 0.014 | **0.015** | -0.081 | 0.019 | **<0.001** | -0.112 | 0.024 | **<0.001** | **(-0.159) -(-0.065)** |
| **(M1) Sleep Quality** |  |  |  | 0.243 | 0.057 | **<0.001** | 0.754 | 0.070 | **<0.001** | **(0.615) -(0.893)** |
| **(M2) Sleepiness** |  |  |  |  |  |  | 0.169 | 0.051 | **<0.001** | **(0.069) -(0.270)** |
| **Age** |  |  |  |  |  |  | -0.018 | 0.019 | 0.340 | (-0.055) -(0.019) |
| **Gender** |  |  |  |  |  |  | -1.229 | 0.489 | **0.012** | **(-2.190) -(-0.268)** |
|  | **R^2^ : .020**  **F (3.559): 3.763, p=.010** | | | **R^2^ : .077**  **F (.558): 11.797, p<.001** | | | **R^2^ : .274**  **F (5,557): 42.208, p<.001** | | | |

a: Obtained by sample of 5,000 Bootstrap

b: 95% Bias corrected Confidence Interval
